# Automated Extraction of Phenotypic Leaf Traits of Individual Intact Herbarium Leaves from Herbarium Specimen Images Using Deep Learning Based Semantic Segmentation

**DOI:** 10.3390/s21134549

**Published:** 2021-07-02

**Authors:** Burhan Rashid Hussein, Owais Ahmed Malik, Wee-Hong Ong, Johan Willem Frederik Slik

**Affiliations:** 1 Digital Science, Faculty of Science, Universiti Brunei Darussalam, Tungku Link, Gadong BE1410, Brunei; 18h8338@ubd.edu.bn (B.R.H.); weehong.ong@ubd.edu.bn (W.-H.O.); 2 Department of Environmental Life Sciences, Faculty of Science, Universiti Brunei Darussalam, Tungku Link, Gadong BE1410, Brunei; johan.slik@ubd.edu.bn

**Keywords:** semantic segmentation, object detection, leaf extraction, connected component analysis, plant species identification, herbarium leaf dataset, phenotypic features, leaf measurements, deep learning

## Abstract

With the increase in the digitization efforts of herbarium collections worldwide, dataset repositories such as iDigBio and GBIF now have hundreds of thousands of herbarium sheet images ready for exploration. Although this serves as a new source of plant leaves data, herbarium datasets have an inherent challenge to deal with the sheets containing other non-plant objects such as color charts, barcodes, and labels. Even for the plant part itself, a combination of different overlapping, damaged, and intact individual leaves exist together with other plant organs such as stems and fruits, which increases the complexity of leaf trait extraction and analysis. Focusing on segmentation and trait extraction on individual intact herbarium leaves, this study proposes a pipeline consisting of deep learning semantic segmentation model (DeepLabv3+), connected component analysis, and a single-leaf classifier trained on binary images to automate the extraction of an intact individual leaf with phenotypic traits. The proposed method achieved a higher F1-score for both the in-house dataset (96%) and on a publicly available herbarium dataset (93%) compared to object detection-based approaches including Faster R-CNN and YOLOv5. Furthermore, using the proposed approach, the phenotypic measurements extracted from the segmented individual leaves were closer to the ground truth measurements, which suggests the importance of the segmentation process in handling background noise. Compared to the object detection-based approaches, the proposed method showed a promising direction toward an autonomous tool for the extraction of individual leaves together with their trait data directly from herbarium specimen images.

## 1. Introduction

Herbarium specimen collections present a unique botanical source of information. They are important data sources for new species discoveries, plant evolution reconstruction, and studying the impact of climate change [1,2,3]. Herbarium plants consist of dried plants with a mixture of damaged, overlapping, and individual intact leaves. Furthermore, these leaves vary in their shape, color, and texture, even within samples from the same species. To create a herbarium sheet, a collected fresh plant undergoes a drying and pressing process that distorts the morphological arrangement of the original plant by folding, overlapping, and placing the leaves at a different position to ensure the specimen fits on a standard herbarium sheet [4].

The current digitization effort of these collections presents both an opportunity and a new challenge for computer vision experts [5]. During the digitization process, additional non-plant objects such as color charts for image quality assessment and a ruler to estimate the physical size of the specimens are added to the sheet [6]. Such items are generally randomly placed in empty places of the sheet to prevent occluding the specimen itself. Hence the final herbarium sheet image contains specimen with folded, overlapping, and single leaves with the addition of other non-plant objects such as color charts, barcodes, rulers, and labels. While these objects are considered useful to botanists and taxonomists, they are treated as noise when applying computer vision techniques to identify certain species [7,8].

Among the plant organs that is mostly present throughout the season, the leaf is considered important as it carries a lot of information regarding the plant [9]. A leaf carries many unique features such as shape, color, and texture, which varies among species and hence makes it a widely used plant organ for different tasks such species identification, species distribution, climatic indicators, and phylogenetic relationships [10,11]. Phenotypical characteristics of the leaves such as leaf length, width, petal size, area and leaf perimeter are important morphological features for the evolutionary studies of plants [12]. Current community efforts to harvest these features is limited as it requires a manual process of analyzing individual specimens that is both time-consuming and a costly operation considering the existing volume of specimens already preserved in herbaria [13]. Existing digitization efforts of these specimens present a great opportunity for the computer vision community to accelerate this process through automation [13]. Studies such as [14] have initiated the process of developing specialized software for extracting phenotypic features although the process is still user-dependent. Full automation of phenotypic trait extraction from digitized herbarium specimens could greatly enhance the existing traits database (such as www.try-db.org, accessed on 20 May 2021) to answer fundamental questions related to biodiversity [15]. Furthermore, extraction of these leaves with their traits could improve the involvement of computer scientists in developing new identification systems for herbarium specimens as most of the existing studies have relied on fresh leaves [16].

In this study, an efficient pipeline (sequence of steps) was proposed to automate the extraction of individual intact leaves together with their phenotypic traits from herbarium specimen images. While these leaves exist in various forms (e.g., damaged, overlapping, and/or intact individual leaves), the proposed method focuses on the extraction of the intact leaves by combining deep learning and image processing techniques. Given a herbarium image, the proposed method automatically localizes and segments intact individual leaves from the rest of the image and extracts morphological measurements from the segmented intact leaves. Specifically, the proposed pipeline consists of three main phases. First, we applied aa deep learning based semantic segmentation model to automatically segment leaves (including damaged, overlapping, and intact individual leaves) from the rest of the objects present in the herbarium sheet. We then enhanced the generated segmentation mask with simple thresholding techniques before applying connected component analysis for localizing candidate leaves. Finally, a simple binary classifier was used to select individual intact leaves from candidate leaves and then extracted the features from the selected leaves. To assess the effectiveness of the proposed method, we compared the proposed method with current state-of-the-art object detection models such as YOLOv5 and Faster R-CNN [17,18]. These methods were also tested on an in-house dataset as well as one of the publicly available datasets [19]. The results obtained showed the robustness of the proposed method.

With the massive investment of both resources and money in the digitization of herbarium collections worldwide, automating phenotypic traits from leaves will improve the utilization of these collections for various biodiversity studies [15]. On the other hand, this will not only increase the value of the herbaria collections but also reduce the cost and time of manually extracting individual leaf images [20]. Effective extraction of individual leaves from herbarium collections will also provide a valuable contribution to botanical research for studies focusing on individual leaves such as [21], thus making better use of the available specimen images. This will also prove important by improving the sample size of the species for studies being conducted in tropical regions where there is a high number of diverse species with highly imbalanced herbarium collection data [5,22]. In summary, the main contributions of this work are as follows:

- We propose an approach to automatically segment and extract phenotypic traits of single intact leaves directly from herbarium specimen images.
- The proposed method has completely automated the task of individual leaf extraction from a given herbarium specimen image without requiring any user intervention.
- We performed experimental validation of the proposed method by comparing its performance with existing state-of-the-art object detection approaches (Faster R-CNN and YOLOv5) on an unseen publicly available dataset (in addition to the in-house dataset) and achieved promising results.
- We curated a new dataset of herbarium specimen images together with their pixel-level ground truth annotation, which can be used for training/testing machine learning techniques.

## 2. Related Works

An important step in automatic phenotypic feature extraction directly from herbarium specimen images is to accurately localize intact leaves. This is important as leaf measurements taken from damaged or non-intact leaves can give misleading results [23]. In most cases, leaf localization is achieved by performing leaf segmentation, which is separating the leaf from the rest of the background. There exist a number of studies that have attempted to segment plant leaves directly from herbarium specimens [24]. These methods are based on active contours [25] using prior shape models [26] and color based methods [27]. These methods perform well when the target leaf has a uniform background but they tend to struggle in the presence of more complex backgrounds such as images with highly variable content. In the case of herbarium specimen images, existing visual noise such as color charts, specimen labels, and other botanical information makes the task of leaf segmentation difficult for traditional segmentation algorithms. On the other hand, deep learning approaches have started to show promising results [13], however, most of these approaches are species dependent and hence do not generalize well on other taxa categories or require large and diverse training samples [12]. 

Corney et al. are among earlier works to attempt automating the segmentation of the leaves directly from herbarium specimens [28]. Their study focused on three species belonging to the genus Tilia L, where they used the canny edge detector algorithm together with a deformable template approach to segment potential leaves from the rest of the objects. In order to extract leaf features, a human expert was required to manually select intact leaves and then perform further processing. Their work was limited as it was not fully automated and techniques such as deformable template were based on prior knowledge of leaf shapes, hence lacking flexibility. Similarly, Henries and Tashakkori proposed using different morphological operations such as opening and closing operations to segment herbarium leaves from their stem [29]. However, their study was performed on a simple experimental setting where the specimen was already isolated from the rest of the objects. 

Recent results on deep learning methods have started to show promising results for the segmentation process. Studies such as [13] attempted to automate the extraction of leaf features using an ensemble of models. The study trained a deep learning semantic segmentation model based on DeepLabv3+ and used a set of selected intact leaves to train a SVM classifier to filter out candidate leaves from the remaining leaves based on leaf length and width. The study involved more than 400 specimen images collected from different herbaria. Furthermore, the authors used a sliding window technique to improve the training sample size and use it as a remedy to downscale the images for training the CNN model while using leaf measurements such as leaf length and width to train a SVM classifier. The study reported an average IoU of 55.2% for the leaf segmentation model on 74 test sets while achieving a recall of 0.98 for detecting at least a single intact leaf from a set of images. In contrast, Ott et al. proposed an object detection technique to automatically identify intact leaves from herbarium specimen images. Their study involved a total of 243 herbarium images mostly from the Leucanthemum species. Their study trained a Faster R-CNN model and reported an accuracy of 95% on a sub-set of 61 test images. Similarly, Younis et al. [30] proposed a Faster R-CNN model to detect and annotate different plant organs from digitized herbarium specimens. The authors manually annotated hundreds of images and used a subset of 498 images to train the model to detect different organs including flowers, leaf, fruit, seed, root, and stem. The study reported an overall average precision (AP) of only 9.7.

Other studies have used similar techniques for digitized herbarium specimens, although they focused on solving different tasks. For example, Abraham et al. [6] applied semantic segmentation to extract herbarium information to assess and automate image quality management. The study aimed to use the segmented information to assess three quality attributes including colorfulness, contrast, and sharpness of the images. Similarly, Hussein et al. [7] proposed using deep learning semantic segmentation techniques to remove background noise in herbarium images. Adán et al. [31] proposed an instance segmentation model for extracting morphological and visual information existing in herbarium specimens. Due to the high demand for annotated datasets for most of the deep learning approaches, the study suggests integrating their model (Mask R-CNN) with an active learning mechanism to minimize the manual annotation process for researchers. Although the study applied instance segmentation in herbarium images, the main focus was to extract visual information such as the number of organs instead of extracting the leaves themselves [31].

A closely related study to this work is the study of Weaver, Ng, and Laport, although our study has made numerous improvements. As discussed, the performance reported by the previous study was relatively small despite a large training sample being used. This is likely caused by training on various class categories apart from leaves only and using a simple feature such as leaf length and width to distinguish intact leaves from non-intact leaves. From the rest of the sections, we show that our approach yielded better results as the segmentation process is more robust to noise than object detection, which is important when extracting botanical features.

## 3. Proposed Methodology

In this section, we provide a detailed explanation of the proposed method by introducing different components of the pipeline. In the next section, the experimental work of the proposed method will be provided. 

**Figure 1:**
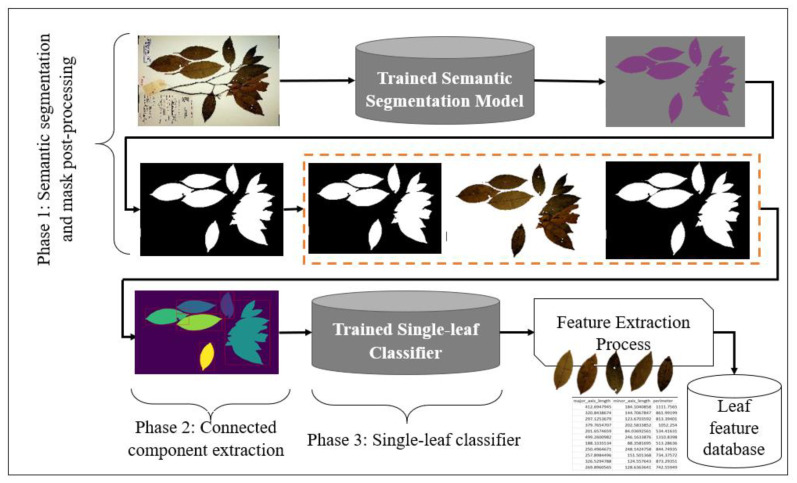
A graphical summary of the single leaf extraction process. In the first phase, the herbarium image is passed through a trained deep learning semantic segmentation model. The generated mask is then enhanced through various image pre-processing techniques before passing the image to the connected component to extract all potential leaves. A trained deep learning classifier based on binary image is then used as a filter to filter out individual leaves from the rest of the detected potential leaves. Finally, phenotypic measurements are then extracted from the filtered individual intact leaves.

The proposed system consists mainly of three phases, as shown in Figure 1. During the first phase (deep learning based semantic segmentation process), the leaves are segmented from the background and the generated mask is enhanced via various post-processing steps. The second phase involves applying connected component analysis for the extraction of components from the output of the first phase, which are the potential leaves. Finally, in the third phase, individual leaves are filtered using a single-leaf classifier trained on binary leaf images.

### 3.1. Phase 1: Deep Learning for Semantic Segmentation

**Figure 2:**
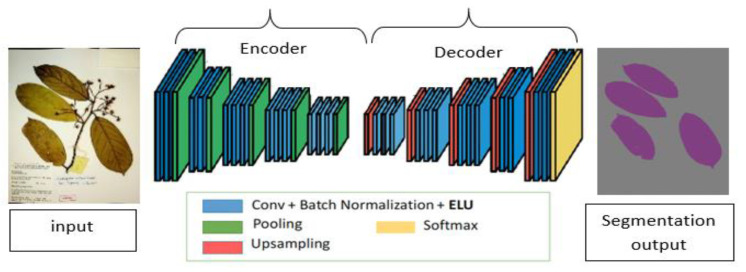
A schematic diagram of a fully convolution neural network for semantic segmentation. The network consists of an encoder part where the model extracts potential useful features and the decoder part, which up-samples the extracted feature map to produce the final segmentation results.

Image segmentation has been a long-term computer vision problem and has been attempted with different algorithms such as image thresholding, Watershed algorithms, Graph partitioning methods, K-means clustering, and many others. CNN’s in image segmentation tasks have received much attention due to its good performance in image classification tasks [32,33]. Segmentation is more challenging as it involves both object detection and localization. This is achieved by assigning labels to each pixel in an image. Semantic segmentation has been widely adapted with either new domain areas of application or improvements in existing architectures [34,35]. Figure 2 shows the basic encoder-decoder architecture of the fully convolutional network used in semantic segmentation tasks. This architecture involves two main parts. The first part is the encoder network t5at uses a modified CNN for classification without the full connected layers to develop a low-resolution feature map of the input with higher efficiency in discriminating between classes. The second part, which is the decoder network, up-samples the learned feature map into a full-resolution segmentation map to provide a pixel-level classification that has the same size as an input image. 

In this phase, we adapted DeepLabv3+ architecture, which follows the same encoder-decoder architecture. This was based on the performance of the DeepLabv3+ model on our previous work related to the segmentation of the whole herbarium specimen [7]. The model has also being widely adapted for herbarium-related studies [36]. Apart from that, DeepLabv3+ has been the state-of-the-art in different benchmarking datasets for semantic segmentation tasks [37]. Deeplabv3+ follows the same encoder-decoder architecture. In the encoder phase, DeepLabv3+ uses pre-trained CNNs that have been trained for image classification tasks such as ResNet or VGG16. DeepLab families uses spatial pyramid pooling to process input images at multiple scales in order to capture multi-scale features and later fuse the output to produce a feature map [38]. To improve its efficiency, an Atrous convolution operation was introduced. This operation enables the window size of the kernel to expand without increasing the number of parameters [39]. This expansion of the window is controlled by the dilation rate and it enables the network to capture information from a larger receptive field of view with the same parameters and computational complexity as the normal convolution. The combination of spatial pyramid pooling with Atrous convolutions resulted in an efficient multi-scale processing module called Atrous spatial pyramid pooling (ASPP). In the earlier version (DeepLabV3) [40], the last ResNet block of the modified ResNet-101 uses different Atrous convolutions with different dilation rates. ASPP, together with bilinear up sampling, is also used on top of the modified ResNet block. DeepLabv3+ is an improvement in the previous version by adding an effective decoder module to improve the boundaries of the segmentation results [41]. Furthermore, apart from ResNet-101, an Xception model can be used as a feature extractor while applying a depth-wise separable convolution to both ASPP and the decoder module, hence improving the speed and robustness of the encoder-decoder network.

### 3.2. Phase 2: Leaf Extraction Using Connected Component

A classic connected component algorithm was first introduced by Azriel and John in 1966 [42]. Since then, numerous different implementations have been proposed for improving existing ones [43]. In image processing, connected components analysis helps to find parts of objects in an image that is physically connected. It works by assigning a given set of pixels a unique label that depends on whether the surrounding pixels are connected or not. Connected-component labelling is necessary for distinguishing different objects in a binary image and has been one of the most important techniques used in image analysis, computer vision, and pattern recognition [44]. Connected component analysis has successfully been used in different domain areas such as leaf vein detection [45], weed detection [46], and character extraction from vehicle plates [47]. In this work, we utilized connected-component labelling for extracting all detected potential leaves, which include overlapping, damaged, and individual leaves from a binary herbarium image obtained in phase 1 of the pipeline.

### 3.3. Phase 3: Single-Leaf Classifier

Herbarium specimens are dried plants that vary in shape, color, and texture even for species belonging to the same taxa. Furthermore, the same sheet can have a mixture of individual leaves, overlapping or damaged leaves, which occurs due to either the preservation process, herbivore activities, physical interaction, or the preservation period. Training a classifier with the normal leaf images will present a great challenge as these categories of leaves share both color and texture. In this way, a large dataset of different categories of leaves (individual, damaged, and overlapping) will be required to train the model with a high probability of poor generalization due to the nature of herbarium leaves themselves.

Due to these constraints, we approached this stage of filtering individual leaves by focusing on the leaf shape only. Given a leaf, we applied pre-processing steps by converting it to a binary image (black and white) and then used the binary image for training. This eliminates the need for large training data but also improves the generalization of the classifier as it specifically focuses on the leaf shape patterns. On the other hand, since different species share similar leaf shapes, a publicly available dataset of individual leaves from fresh plants could be used as a training sample as they require minimum pre-processing. In this phase, we adapted the VGG16 network architecture with few modifications, which will be explained in later sections. With this approach, the proposed method is more flexible and can deal with different leaf shapes, color, and even herbarium leaves with small deformations. Earlier studies have limited the classifier by considering the leaf size as a feature [13]. As the study showed, they require a large training sample to maintain a good performance of their approach.

## 4. Experimental Work

In this section, we introduce the experiment performed for developing the proposed method. All the experiments were carried out on a machine equipped with an Intel i7 8th generation CPU, 16 GB RAM together with NVIDIA GeForce GTX 1060 Max-Q Design in a 64-bit Windows 10 environment.

### 4.1. Datasets

In this study, we used three different datasets. The first dataset included herbarium sheet images collected from the in-house herbarium (UBDH). This dataset was used for training and evaluation of the semantic segmentation model. The second dataset was the Herbarium Challenge 2019 Dataset (HCD) [19], which is publicly available. This dataset was used to evaluate and validate the performance of the proposed method. The third dataset was a combination of individual herbarium leaves together with a subset of the Flavia dataset. The Flavia dataset [48] (publicly available) consists of individual leaves with the blade only on a plain background. This dataset was used as part of training a single-leaf classifier.

**Figure 3:**
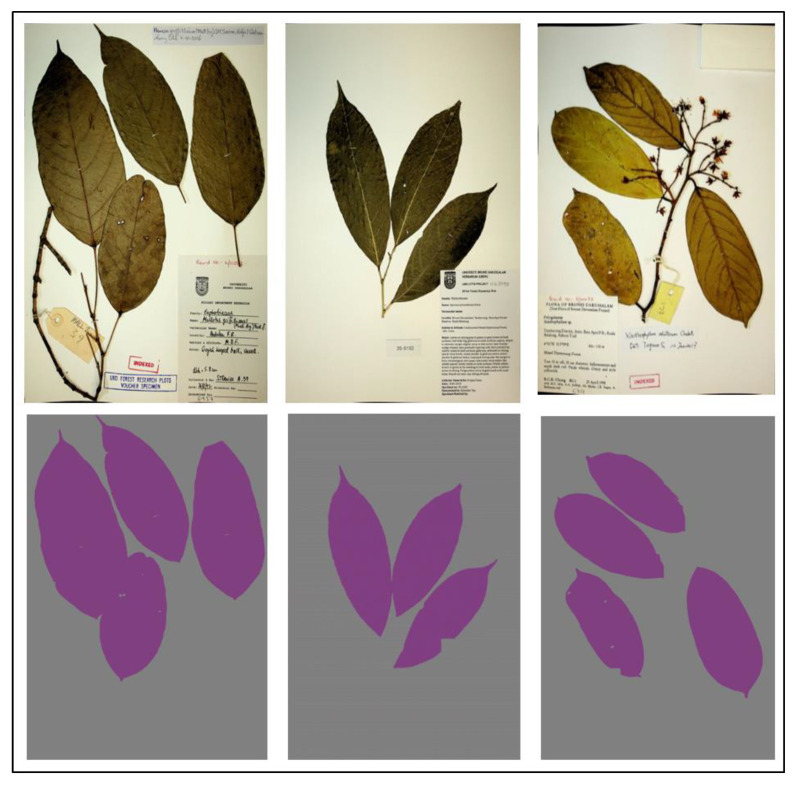
A sample of herbarium images (**top** row) and their corresponding annotation (**bottom** row) used for training the segmentation model from the UBDH dataset. This dataset consisted of 500 herbarium images together with their ground truth annotation for the training segmentation model.

UBDH Dataset—This dataset consists of 500 herbarium images together with their annotations (ground truth) for training the segmentation model. The UBDH dataset contains more than 8000 plant species from a tropical region and is currently undergoing digitization. The image labeler app from MATLAB 2018 software was used to generate the ground truth labels. We then applied a median filter to reduce any noise that may have been introduced during the labelling process. Figure 3 shows an example of herbarium images and their ground truth labels. After the labelling process, the dataset consisted of two classes: leaves and background. We later converted the dataset into the coco dataset format and made it publicly available for future research purposes. 

HCD Dataset—This dataset contains herbarium images of the flowering plant family Melastomataceae [19]. We randomly selected a subset of this dataset containing 90 herbarium images with at least an individual leaf that can be extracted for evaluating the proposed method. This dataset aimed to assess the generalizability of our proposed method and compared it with existing state-of-the-art approaches.

Single-leaf Classifier Dataset—To train a single-leaf classifier, we utilized two different datasets and combined them. The first dataset was the herbarium leaves. This dataset was generated by passing the same training data used for the semantic segmentation model (phase 1) and later used a connected component to extract all the detected leaves (phase 2). Finally, we manually separated these leaves into individual (leaves having recognizable outer shape or margin) versus non-individual leaves (i.e., damaged leaves, partial leaves, and overlapping leaves).

**Figure 4:**
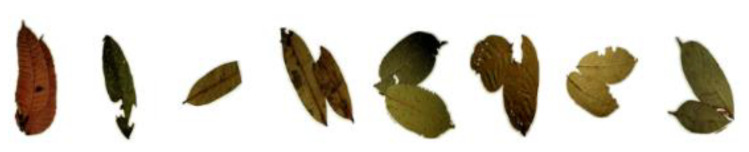
Negative samples manually extracted from the segmentation model results for training the single-leaf classifier. The dataset consisted of 881 intact individual leaves as positive training samples and 1015 negative samples.

To improve the generalization of our classifier, we used a subset of the Flavia dataset containing 83 individual leaves samples of 32 different species. The selected leaves had a similar shape to the herbarium leaves. The combination of the two datasets was important to improve the robustness of the classifier when presented with a new dataset. A summary of the dataset used is given in Table 1. Figure 4 shows some of the negative samples used for training the classifier.

### 4.2. Pre-Processing and Training of Semantic Segmentation Model

Herbarium sheet images are usually of high resolution to capture the fine-grain details of the specimens. As a standard procedure for training deep learning models, all input images together with their annotation were resized to a 512 × 512 resolution to reduce the computational cost during training. Rotation, flipping, and brightness adjustments were applied as augmentation techniques for better network generalization. We used DeepLabv3+ as the segmentation model with ResNet-101 as the feature extractor. This model was pre-trained on the ImageNet dataset and fine-tuned on the dataset. This is useful as earlier layers of the network tend to learn generic features and therefore become useful for other computer vision tasks [49]. We applied an Adam optimizer with a learning rate of 1 × 10^−4^ and a batch size of 3. The model was trained for 100 epochs with a binary cross-entropy loss function as we had a binary class problem (leaf or background).

**Figure 5:**
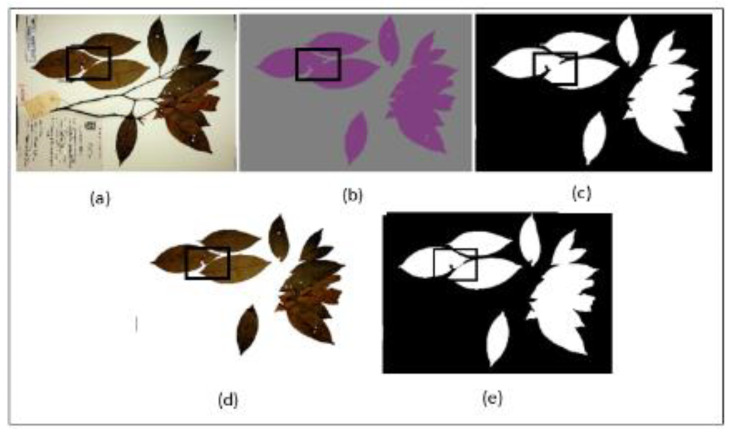
Mask post-processing step, (**a**) original image, (**b**) generated mask from segmentation model, (**c**) image b after thresholding, (**d**) new image after masking operation between image a and c, (**e**) new generated mask to be passed to phase 2.

Mask Post-processing—Although our segmentation model successfully segmented between the leaves and the background, there exists a tendency of the model to under-segment individual leaves that were close together within a single mask. Figure 5b shows an example of the model output with the under-segmentation of closely placed leaves highlighted with a black square box. This presents a challenge as most of the individual leaves are closely placed due to the limitation in the size of the herbarium sheet. To solve this problem, simple post-processing steps were applied.

First, the generated mask from semantic segmentation model was resized to the original high-resolution dimension of the herbarium images. This is a vital step as we wanted to extract fine details of the leaves in the later stages without sacrificing image quality, hence, we only resized the mask and not the herbarium image itself. In the next step, the generated mask was converted to a binary mask using Otsu thresholding [50] followed by a 5 × 5 dilation (5 × 5 kernel was determined as the best size after several experiments). The dilation operation helped in ensuring the margins of the leaves were covered from the generated mask. In the third step, a flood-fill operation was applied to cover the holes that were present inside the margin of the leaves (Figure 5c). This operation helped in preventing any artifacts being introduced when applying the masking operation between the current mask and the original herbarium image again (Figure 5d). Since the new image had a clean background with distinct color between the leaves and the background with the clear boundary between closely placed individual leaves, Otsu thresholding was again applied. Finally, another flood-fill operation was applied to the segmented image to ensure the mask covered the whole leaf, which may have been missed by the thresholding process (Figure 5e). This step is also important to ensure that the whole leaf is detected when applying connected component analysis in the next step. The output of this step is a clean mask which is then taken to phase 2. The summary of the whole process is explained in Algorithm 1 below.
**Algorithm 1**. Mask Post-processingInput: Herbarium image *H_i_*, mask *m*Output: New herbarium mask *m_n_*1:   begin2:    *m_s_* ← Resize m to the same size as *H_i_*;3:    *m_o_* ← Apply inverse_otsu_threshold to *m_s_*;4:    *m_f_* ← Apply flood-fill operation to *m_o_*;5:    *m_d_* ← Dilate *m_f_* by 5 pixels;6:    *H_n_* ← Apply masking operation between md and *H_i_*;7:    *m_i_* ← Apply inverse_otsu_threshold to *H_n_*;8:    *m_n_* ← Apply flood-fill operation to *m_i_*;9:   end

### 4.3. Leaves Extraction Using Connected Components

The connected component analysis was applied to the binary image generated from the mask post-processing step (Figure 1: phase 1) to extract different components (potential leaves). Since it is well known that herbarium images are of high resolution, we only considered components that had an area greater than 1000 pixels as most of the components with a smaller area were found to be noise. For each of the detected components, a mask was generated and dilated by 10 pixels (obtained best after a number of experiments), which considered only that area of the component as active while ignoring the rest of the image. This step was necessary to prevent nearby components being extracted together with the current component. Subsequently, the bounding box coordinate, which covers the whole component, was used to extract the component from both the component mask and the relevant part from the original herbarium image followed by the masking operation between the two. The masking process was important to generate a leaf image with a clean background by removing any nearby leaf that was covered in the bounding box coordinates. The output of this step (Figure 1: phase 2) is then passed to the single-leaf classifier for filtering whether the component detected was an individual leaf or not. The algorithm summarizing the extraction process is presented below (Algorithm 2).
**Algorithm 2.** Extracting Detected ComponentsInput: Herbarium image *H_i_*, New herbarium mask *m_n_*Output: A set *C* of extracted components *c_x_*1:   *C*
*← Ø*;2:   call ← Detect all component *c_s_* in *m_n_*;3:   N ← Find the total number of detected components in call;4:   for *c_s_* ← 1 to *N* do5:    if Area (*c_s_*) > 1000 pixels6:     *m_cs_* ← Create a mask of the current *c_s_* only from *m_n_*;7:     *m_cd_* ← Dilate *c_s_* in *m_cs_* by 10 pixels;8:     *H_cd_* ← Apply masking between *m_cd_* and *H_i_*;9:     *c_x_* ← Crop the bounding box of *c_s_* from *H_cd_*;10:    *C* ← *C* ∪ {*c_x_*};11:    end if12:   end for

### 4.4. Pre-Processing and Training of Single-Leaf Classifier

**Figure 6:**
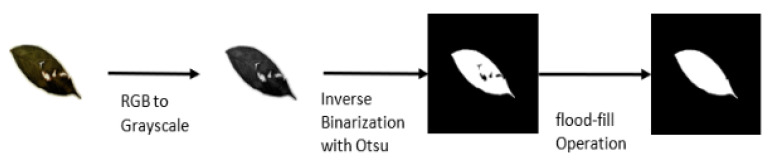
Pre-processing steps for training a single-leaf classifier. For each training sample, the image is converted to grayscale and binarized using the Otsu algorithm. Finally, a flood-fill operation is applied to fill in the missing pixel in-side a binary leaf.

To preprocess the dataset, first, we padded all images with extra pixels to achieve a square (1:1) aspect ratio to ensure maintaining the shape of the leaves while resizing the images before training. Extra padding is also important for data augmentation during the training process to prevent individual leaf shapes from being distorted. The image was then converted to grayscale followed by inverse Otsu thresholding due to the white background of the images. Furthermore, a flood-fill operation was then applied to fill in any holes existing inside the leaves. This operation was performed to extract not only damage-free leaves but also damaged leaves with a recognizable outer shape. Figure 6 summarizes the pre-processing step. 

Training procedure—We adapted a pre-trained VGG16 network that is a CNN trained on the ImageNet dataset and used for transfer learning on our dataset [51]. We froze earlier layers of the base version of the network to make them non-trainable and added an extra max-pooling layer before the fully connected layers to reduce the dimension of the previous layer. The feature vector of the fully connected layer was reduced from 2048 units of the original VGG16 to 128 units, which helped in reducing the computational complexity without sacrificing much on performance. The model was implemented using Keras with TensorFlow backend [52].

We trained with a batch size of 32 images per iteration and applied binary cross-entropy as the loss function. All input images were resized to 300 × 300 resolution and trained for 100 epochs with an Adam optimizer at a learning rate of 1 × 10^−4^. We also applied data augmentation for the training images such as flipping and rotation, with height and width shift as leaves were expected to be indifferent orientations, size, and location hence helped the model to generalize better. The trained classifier was then used as a filter to detect whether the detected component was an individual leaf or not. This process is summarized in Algorithm 3.
**Algorithm 3.** Filtering Individual LeavesInput: A set *C* of extracted components *c_x_*Output: Set *L* of filtered individual leaves1:   *L*
*← Ø*;2:   call ← Detect all component *c_s_* in *m_n_*;3:   N ← Find the total number of components in *C*;4:   for *c_x_* ← 1 to *N* do5:    *c_p_* ← Apply pre-processing steps;6:    flag ← Pass *c_p_* to a trained single_leaf_classifier;7:    if flag = = leaf8:     *L*
*← L* ∪ {*c_x_*};9:    end if10:   end for

Table 2 provides a summary of the hyperparameters used for the semantic segmentation model and the single-leaf classifier. We used a larger input dimension for the case of the segmentation model since herbarium images are of high dimensions (usually 3000 × 2000 or more). For the case of a single-leaf classifier, we reduced the input dimension as we were only dealing with the extracted components with a binary image.

### 4.5. Comparison with the State-of-the-Art Approaches

To assess the performance of the proposed method, we compared it with the current existing state-of-the-art object detection techniques such as Yolo architectures and Faster R-CNN network [53,54]. For the YOLO architecture, we adapted the recently released YOLOv5, which has made significant improvements over its predecessors [17], while for Faster R-CNN architecture, we used the implementation available in the detectron2 framework for training with our custom dataset [18]. Training setup for each architecture is as follows:

Faster R-CNN network: For the Faster R-CNN network, we used the publicly available implementation using the detectron2 framework. Since the Faster R-CNN network is a multi-stage detection model (two-stage detector), the network consists of a feature pyramid network (FPN) as a backbone that has a multi-scale pyramid convolutional structure to perform multiscale feature extraction. The extracted features were then used as input to a region proposal network (RPN) to propose multiple regions with objects. Finally, a Fast R-CNN network was used as a head to detect multiple objects. A detailed description of the network can be found in [18]. In this study, we performed a fine-tuning of the pre-trained Faster R-CNN network, which was trained on a MS COCO dataset for object detection task. The network was trained with a batch size of 2, a stochastic gradient descent (SGD) optimizer with a learning rate of 0.00025, and used a 0.6 non-maximum suppression (NMS) threshold during training for 3000 iterations. At the end of the training process, the best performing model based on the validation loss was saved and used as the Faster R-CNN model with a NMS threshold of 0.7 during testing.

**Figure 7:**
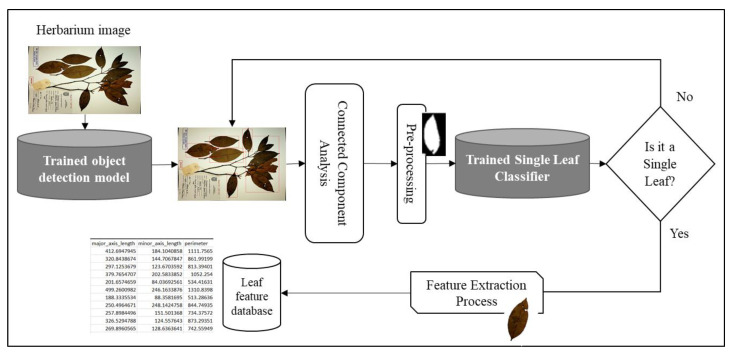
Object detection-based approach for single leaf and feature extraction. The same setup was used as the proposed method except that the segmentation process was replaced by an object detection approach.

YOLOv5s: Unlike Faster R-CNN, YOLO architectures belong to the family of single stage detectors that enable a fast end-to-end network training and inference time. Since after the first release of YOLO architecture, newer versions have focused on incremental improvements in areas including backbone feature extractors such as cross stage partial networks, network training strategies with novel augmentation methods such as mosaic data augmentation, incorporating different training losses such as complete intersection over union (CIoU-loss) and focal loss to address the imbalance between the foreground and background classes, activations such as Mish activation, and other universal feature extraction strategies such as cross-stage-partial-connections (CSP), weighted-residual-connections (WRC), etc. [55]. In this work, we utilized the recently proposed YOLOv5s architecture. YOLOv5 architecture shares many similarities with its YOLOv4 counterpart, nevertheless the authors of YOLOv5 have automated the process of anchor box selection by learning the bounding box distribution of a new dataset using k-means and genetic algorithm and hence making the network easily adaptable to train with other datasets [17]. We also performed a fine-tuning process of YOLOv5s, which was trained from the MS COCO dataset for the object detection task. The network was trained for 300 epochs using an Adam optimizer with an initial image size of 640 × 640 and a batch size of 16. Like in the Faster R-CNN training process, we stored the best performing model based on validation loss and used it for inference. To improve the network results, we used test time augmentation with a 0.6 NMS threshold. Both networks were trained to detect potential candidate leaves from the herbarium images using the same train/validation and test dataset as the one used to train the proposed segmentation model. For each detected potential leaf from the networks, an Otsu thresholding and flood-fill operation was applied before using connected component analysis to extract the largest component. The extracted largest component was then passed to a single-leaf classifier to detect whether the object was an individual intact leaf or not. This pre-processing improved the performance of the single-leaf classifier as the classifier was trained with a binary leaf image. Figure 7 depicts the approach used for the object detection-based method for individual leaf extraction. The correctly classified intact leaves were then used to extract various phenotypic features. 

## 5. Performance Evaluation Metrics

The procedure used in evaluating the proposed method versus other approaches was conducted as follows. We selected herbarium images that consisted of at least a single individual leaf. In this way, we were able to count how many leaves were extracted by the proposed method against how many leaves we expected. This stage helped us to investigate the effectiveness of each approach in the extraction of an individual leaf. We selected a total of 144 herbarium images, 54 from the UBDH dataset, and 90 images from the HCD dataset. From the UBDH dataset, we expected to extract a total of 190 individual leaves while in the HCD dataset, we expected to extract a total of 260 individual leaves after manually inspecting the images. None of these selected images were used in the training stage.

For performance evaluation, different metrics were used to evaluate the performance of the individual models as well as of the whole pipeline on individual leaf extraction. Mean intersection over union (MIoU) is a standard metric for evaluating segmentation models [56]. It provides an average score of all classes by quantifying the overlap between the target label and the predicted label. MIoU is calculated by taking the ratio of true positives over the sum of false positives, false negatives, and true positives. Equation (1) shows how to calculate the MIoU, which is calculated pixel-wise in the case of segmentation model. We also adapted different performance metrics such as accuracy, precision, recall, and F1 score to assess the performance of the proposed system (Equations (2)–(5))

(1) $$\mathrm{MIoU}\;=\frac{1}{N}\overset{N}{\underset{x=1}{\sum }}\;\frac{N_{\mathrm{xx}}}{\sum_{y=1}^{N}{\;N}_{\mathrm{xy}}+\sum_{y=1}^{N}{\;N}_{\mathrm{yx}}-{\;N}_{\mathrm{xx}}}$$

(2) $$\mathrm{Accuracy}\;=\frac{N_{\mathrm{xx}}+{\;N}_{\mathrm{yy}}}{N_{\mathrm{xx}}+{\;N}_{\mathrm{yy}}+N_{\mathrm{xy}}+{\;N}_{\mathrm{yx}}}$$

(3) $$\mathrm{Precision}\;=\frac{N_{\mathrm{xx}}}{{\;N}_{\mathrm{xx}}+N_{\mathrm{xy}}}$$

(4) $$\;\mathrm{Recall}\;=\frac{N_{\mathrm{xx}}}{{\;N}_{\mathrm{xx}}+{\;N}_{\mathrm{yx}}}$$

(5) $$\;F1\;\mathrm{score}\;=\frac{2\times {\;N}_{\mathrm{xx}}}{\;2\times {\;N}_{\mathrm{xx}}+N_{\mathrm{xy}}+N_{\mathrm{yx}}\;}$$

where N is the total number of classes; ${\;N}_{\mathrm{yy}}$ is the true negative; $N_{\mathrm{yx}}$ is the false negative; $N_{\mathrm{xx}}$ is the true positive; and $N_{\mathrm{xy}}$ is the false positive. For object detection models, we used a mean average precision metric (mAP) to measure the overlap between the predicted bounding box against the ground truth or the predicted pixels against the ground truth pixels for the segmentation task. We also used other metrics such as mean absolute error (MAE), mean square error (MSE), and root mean square error (RMSE) to measure how well the extracted phenotypic traits matched those of the ground truth features.

## 6. Results and Discussions

The results section presents the performance evaluation of the semantic segmentation model and single-leaf classifier along with a comparison between the proposed method and the object detection-based approaches for individual leaf extraction from herbarium specimen images.

### 6.1. Evaluation of Semantic Segmentation Model

We divided the UBDH dataset into 80% training, 10% validation, and 10% for testing. The model achieved an average accuracy of 95.59% on 57 test samples. Table 3 summarizes the results of the two-class semantic segmentation model used in the proposed method for differentiating the leaf and the background. For the leaves class, the model achieved an accuracy of 92.21% on testing samples that was slightly lower than the background class, which achieved an accuracy of 98.98%. This difference between the two accuracies may suggest that the model has a certain degree of under-segmentation of the leaves, which was corrected by the mask post-processing step after the segmentation process. Nevertheless, our model achieved a satisfactory result with a MIoU of 94.17% and 93.71% in the validation and testing sets, respectively. On the other hand, the performance of the YOLOv5s model seems to be higher than both DeepLabv3+ and Faster R-CNN in terms of leaf localization on a test set (Table 4) but as shown in the next section, this model does not generalize well presented with a new dataset. The results of the segmentation model suggest that DeepLabv3+ architecture is more efficient to work with a relatively small dataset compared to most of the deep learning implementations while maintaining good generalization on a new dataset.

### 6.2. Evaluation of Single-Leaf Classifier

**Figure 8:**
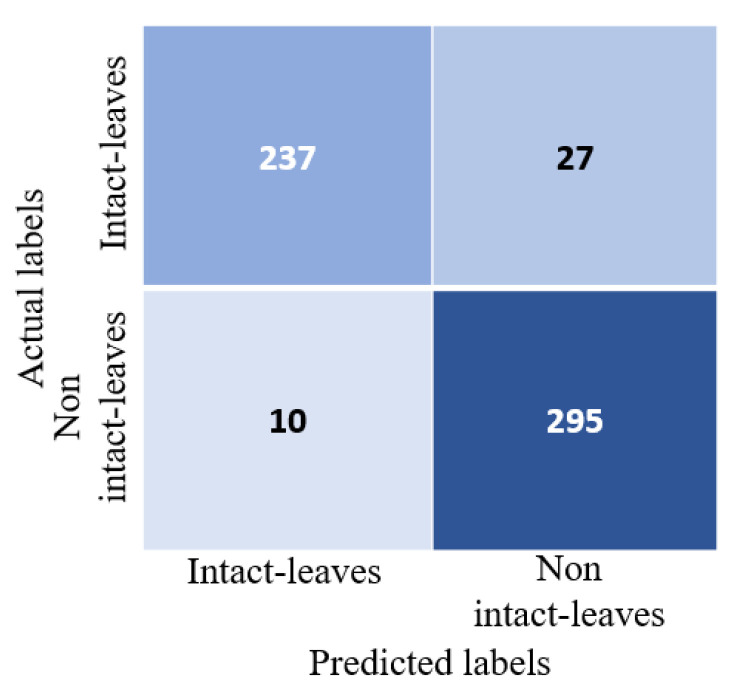
Confusion matrix for a single-leaf classifier on a separate test set. The test set consisted of 264 individual intact leaves (positive samples) and 305 non-intact individual leaves (negative samples).

To train the classifier, the single-leaf classifier dataset was divided into 70% training and the remaining 30% for testing. The classifier achieved a testing accuracy of 93.49% with an area under curve score (AUC) of 93.24%. Figure 8 shows the confusion matrix for the test set. From the confusion matrix, we can see that the classifier had a slightly higher precision of 93.62% than recall, which was 93.5%. It also shows that the model made few mistakes in classifying non-individual leaves as individual leaves than classifying individual leaves as non-individual leaves. This is perhaps an acceptable mistake when we want to automate the extraction of features using the proposed method as the model would not miss many samples of individual leaves. 

### 6.3. Evaluation of the Proposed Method for Single Leaf Extraction

**Figure 9:**
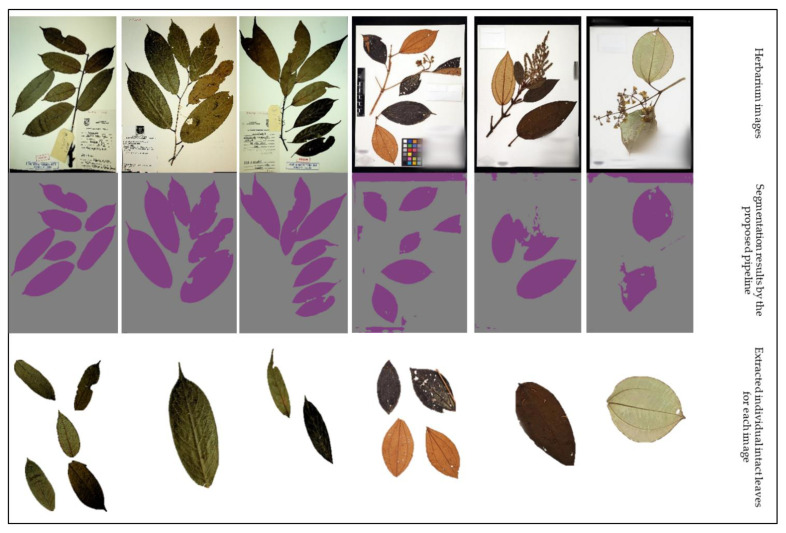
Samples from the UBDH and HCD evaluation datasets (**top row**) together with the predicted segmentation mask using the proposed method (**middle row**). The bottom row represents intact individual leaves that the proposed method was able to extract. The first three columns represent evaluation samples from UBDH (consisted of 54 image samples) and the last three columns represent evaluation samples from the HCD (consisting of 90 image samples).

**Figure 10:**
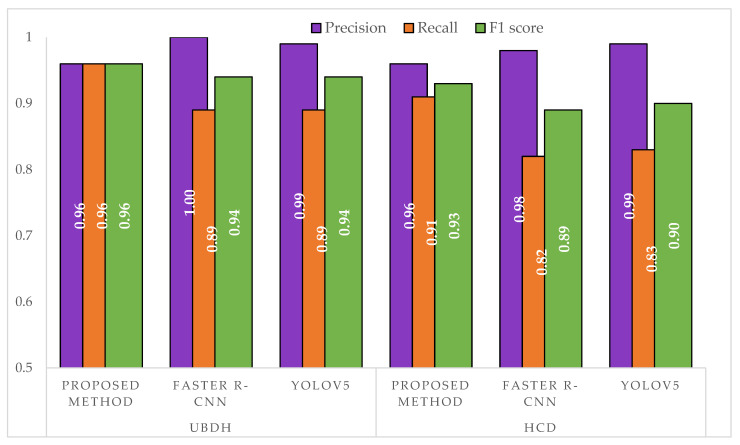
Comparison of precision, recall, and F1 score between the approaches on a separate test set. The UBDH dataset consisted of 54 images with a total of 190 individual intact leaves. The HCD dataset consisted of 90 images with a total of 260 individual leaves.

**Figure 11:**
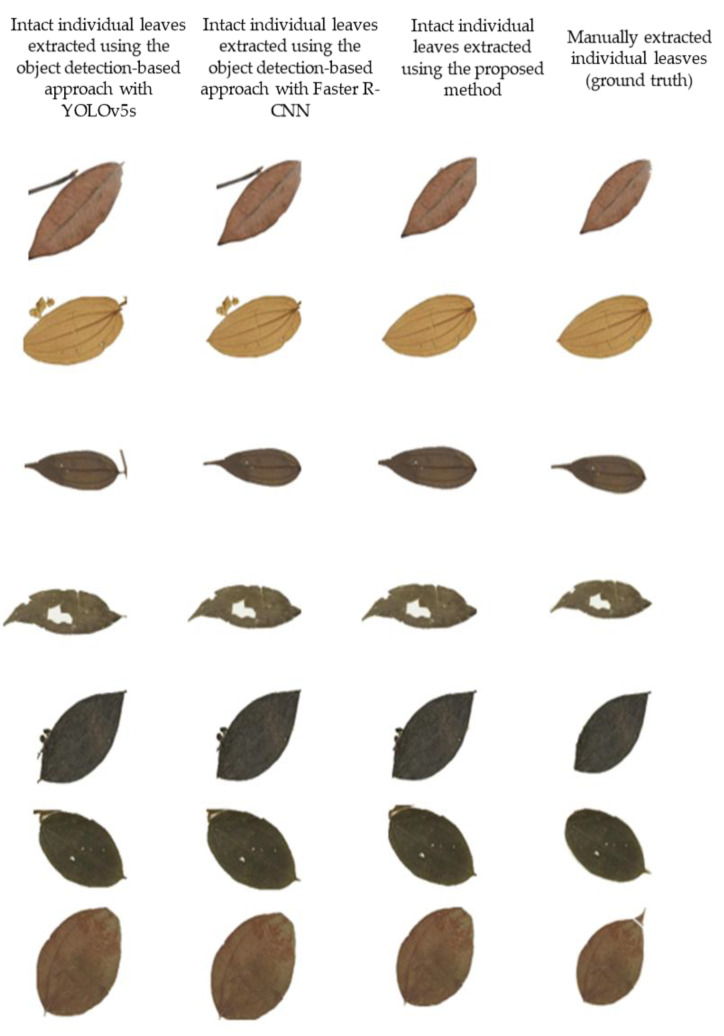
Samples of intact leaves extracted and used for feature extraction. First row represents the ground truth leaves that were manually segmented; the second row consists of leaves extracted using the proposed method; the third row represents leaves extracted based on the Faster R-CNN model; and the last row represents the leaves extracted when using the YOLOv5s model. The first three columns for object detection-based approaches showed some artifact encountered when using object detection-based approaches as opposed to segmentation. The last three column showed some failure cases even for the segmentation model with small artifacts at the boundary of the leaves and missing leaf apex.

From the results in Table 5, we can see that by using the proposed method, we were able to extract a total of 175 individual leaves from the UBDH dataset and a total of 256 individual leaves from the HCD dataset. A sample of these images can be seen in Figure 9. The proposed method achieved a high precision and recall in both datasets (Figure 10) that showed a good generalization when presented with a new dataset such as HCD, which was not used in any part of the training. In contrast, both Faster R-CNN and YOLOv5s approaches achieved a higher precision than the proposed method but suffered in the recall, which had a high number of false negatives. This may be attributed due to the fact that the images that were passed to the single-leaf classifier using the object detection-based approach contained leaf with other parts of the plants (such as stem attached to the leaf), causing the classifier to classify them as a non-individual leaf. As shown in the feature extraction part, even for the correctly identified individual intact leaves, the object detection-based approaches introduced artifacts that may hinder precise the trait extraction process. Figure 11 shows some of the output from the intact leaf extraction pipeline. 

From the results in Table 5, it can be seen that the semantic segmentation model plays a crucial role in the effective extraction of individual leaves as the proposed method only failed to recognize 19 leaves out of all possible leaves, which matches the performance of the Faster R-CNN approach (Table 6).

However, as shown in Figure 11, the output of the proposed method could effectively deal with noise along the leaf, hence making it desirable for automating the feature extraction process. Most of the failure cases by the proposed method may be due to under-segmentation or over-segmentation of the leaf, hence failing to capture the proper leaf shape. With the object detection-based approaches, the number of undetected leaves was high for the YOLOv5s as more than 7% of the leaves were not detected (Table 7). On the other hand, since the images extracted with the object detection approach were not as clean as the one in the proposed method, a classifier needs to be much more robust to detect the leaf when using this approach. Figure 11 shows a sample of the individual leaves extracted using all the approaches. With the current volume and size of herbarium images, the proposed method seems to be much more efficient and robust even when using a simple shape-based classifier for filtering individual leaves from other leaves. Table 8 presents a side-by-side comparison between the proposed method based on semantic segmentation against similar approaches when using object detection. As shown in Table 8, using semantic segmentation, the proposed method was able to extract more intact leaves than the object detection approaches. However, using the segmentation model resulted in a higher false detected individual intact leaf. Nevertheless, the proposed approach had a lower number of misclassified individual intact leaves (false negative), which suggested that most of the individual leaves present on the herbarium images were correctly segmented. 

### 6.4. Phenotypic Trait Extraction Process

To assess the quality of the individual intact leaf extraction process, we further extracted a number of leaf traits commonly used for species identification [57,58]. We manually segmented 76 intact leaves from the HCD dataset and extracted the features (ground truth). We then compared these traits with the one extracted by the proposed approach. The intuition behind is that, if these features are close to the ground truth features, it means that the segmentation process from the proposed method is important for the precise trait extraction process. In contrast, if the features obtained by object detection approaches are better than the proposed method, this suggests that using object detection approaches with connected component analysis can produce a precise feature and would be more desirable than an expensive segmentation process.

As illustrated by the results in Table 9, features extracted by the proposed approach were much closer to the ground truth features than the other approaches. For example, when looking at the MAE of features such as leaf area, there is a large deviation for features extracted with object detection approaches. The same can be observed while looking at the other features, which suggests that the proposed segmentation process is much more robust in handling noise that the other approaches. Figure 11 shows some of the failure cases between the proposed approaches. It is clear that other approaches suffer when the leaf is overlapped by another object such as the plant stem. However, all the approaches including the proposed approach have difficulties when dealing with tapped leaves as it only detects the majority part of the leaf and miss the leaf apex. Although the situation is not always present in all herbarium images, further research is required to ensure that the whole leaf is accurately segmented including the tapped region of the leaf.

## 7. Conclusions and Future Work

From the reported results, it can be concluded that the proposed semantic segmentation-based approach for the extraction of individual intact leaves is much more efficient and accurate than the existing object detection approaches. This method has four benefits: (1) the use of the semantic segmentation model enables the extraction of individual leaves even while using a weak classifier trained on a binary image with a small dataset; (2) the semantic segmentation model used in the proposed method can be utilized as a pre-processing step for removing visual noise that exists in herbarium specimens before applying classification algorithms as used in [7] or performing feature extraction compared to object detection-based approaches; (3) the extracted leaves had a uniform white background, which could be an advantage for pre-processing tasks such as segmentation for feature extraction as shown in the result section; and (4) using the proposed method, it becomes possible to automatically extract individual leaves directly from herbarium specimen images. As opposed to the proposed method, object detection-based approaches can offer a simple solution for the location and extraction of leaves when the target task does not require precise leaf information such as phenotypic extraction of features from an individual intact leaf.

This is an important step toward full utilization of existing digitized herbarium collections and for new studies that intend on examining individual leaves. The generated datasets together with the extracted traits will be useful in applying other feature extraction techniques for building automated species identification systems. This will also be a useful step toward developing cross-domain identification systems that involve both fresh leaves and dried leaves from the herbarium specimens where the extraction of features from individual intact leaves could be important. Furthermore, both the filtered individual leaves and non-individual leaves (overlapping and damaged leaves) can be processed for developing plant species identification systems that may provide a more practical utilization of the specimens than the existing identification systems that only expose the center of the image to the classifier or feed the whole herbarium image.

The proposed method can be easily extended to extract other plant organs from the specimen such as flowers or fruits, which could be important for other studies. Moreover, the performance of the overall pipeline can be improved by improving the models used in each phase. In future work, we intend to explore different techniques in dealing with overlapping leaves as well as damaged and taped leaves, which are also common in herbarium specimens. We also intend to further extract different features from the extracted leaves for building a species identification system.

## Figures and Tables

**Table 1:** Dataset summary used for training the single-leaf classifier.

Datasets	Single Leaves (Positive Samples)	Non-Single Leaves (Negative Samples)
UBDH dataset	798	1015
Flavia dataset	83	0
Total	881	1015

**Table 2:** A summary of the hyperparameter used for training the deep learning models.

	Segmentation Model	Single-Leaf Classifier
Input dimension	512 × 512	300 × 300
Batch size	3	32
Learning rate	1 × 10^−4^	1 × 10^−4^
Optimizer	Adam optimizer	Adam optimizer
Loss function	binary cross-entropy	binary cross-entropy
Epochs	100	100
Pre-trained network	ResNet101-DeepLabv3+	Modified-VGG16

**Table 3:** Performance of semantic segmentation model.

Performance Metric	Validation Results	Testing Results
Leaf Acc	92.87%	92.21%
Background Acc	99.71%	98.98%
MIoU	94.17%	93.71%

**Table 4:** Performance comparison of all models on the test set.

Model	mAP	Precision	Recall
DeepLabv3+	95.6	98.9	98.3
YOLOv5s	99.8	100.0	100.0
Faster R-CNN	88.2	88.2	90.9

**Table 5:** Performance of the proposed method for single leaf extraction.

Dataset	No of Images	TP	FP	FN	Total Expected	Total Extracted	Undetected Leaf
UBDH	54	168	7	7	190	175	15
HCD	90	232	10	24	260	256	4

**Table 6:** Performance of Faster R-CNN for single leaf extraction.

Dataset	No of Images	TP	FP	FN	Total Expected	Total Extracted	Undetected
UBDH	54	161	0	19	190	180	10
HCD	90	206	4	45	260	251	9

**Table 7:** Performance of the YOLOv5s approach for single leaf extraction.

Dataset	No of Images	TP	FP	FN	Total Expected	Total Extracted	Undetected
UBDH	54	157	1	19	190	176	14
HCD	90	201	2	41	260	242	18

**Table 8:** Summary comparison between the proposed method (based on semantic segmentation) vs. object detection-based approaches. ^↑^ indicates that a higher value reflects better performance, ^↓^ indicates that lower values reflect better.

	UBDH			HCD		
Metrics	Proposed Method	Faster R-CNN	YOLOv5s	Proposed Method	Faster R-CNN	YOLOv5s
TP ^↑^	168	161	157	232	206	201
FP ^↓^	7	0	1	10	4	2
FN ^↓^	7	19	19	24	45	41
Total extracted ^↑^	175	180	176	256	251	242
Undetected leaf ^↓^	15	10	14	4	9	18

**Table 9:** Comparison of leaf measurement differences against the ground truth for 76 manually collected leaves. For all metrics, lower values indicate better results.

	MAE			MSE			RMSE		
	Proposed Method	Faster R-CNN	YOLOv5s	Proposed Method	Faster R-CNN	YOLOv5s	Proposed Method	Faster R-CNN	YOLOv5s
eccentricity	**0.0030**	0.0044	0.0038	**0.0000**	0.0001	0.0001	**0.0049**	0.0093	0.0073
area	**342.6184**	455.3947	1289.6842	**162,057.5526**	369,695.8158	26,847,791.5395	**402.5637**	608.0262	5181.4855
bbox_area	**691.2632**	1093.0789	2700.8289	**1,193,423.7632**	2,783,964.2368	60,037,639.8816	**1092.4394**	1668.5216	7748.3960
convex_area	**361.5132**	628.8816	1511.9474	**230,122.8816**	1,131,211.3289	29,410,001.1579	**479.7112**	1063.5842	5423.0989
equivalent_diameter	**1.2161**	1.6611	3.9151	**1.9709**	5.7467	206.0384	**1.4039**	2.3972	14.3540
extent	**0.0099**	0.0123	0.0174	**0.0002**	0.0003	0.0005	**0.0143**	0.0185	0.0225
filled_area	**343.9474**	461.2895	1300.0921	**164,526.7895**	383,162.7632	27,113,134.6711	**405.6190**	619.0014	5207.0274
major_axis_length	**1.6697**	2.8865	5.6320	**6.1220**	40.6365	349.0719	**2.4743**	6.3747	18.6835
minor_axis_length	**1.1301**	1.4556	3.3250	**1.5845**	6.0962	130.0219	**1.2588**	2.4690	11.4027
perimeter	**10.0381**	21.1247	33.1226	**258.5453**	1608.1119	4932.9094	**16.0793**	40.1013	70.2347
solidity	**0.0049**	0.0077	0.0081	**0.0000**	0.0005	0.0004	**0.0068**	0.0227	0.0204
diameter	**1.2207**	1.6507	3.9047	**1.9746**	5.6350	205.8782	**1.4052**	2.3738	14.3485
aspect_ratio	**0.0189**	0.0330	0.0288	**0.0008**	0.0080	0.0042	**0.0288**	0.0894	0.0645
rectangularity	**0.0019**	0.0035	0.0046	**0.0000**	0.0002	0.0002	**0.0030**	0.0145	0.0128
compactness	**0.5694**	1.0373	1.2096	**0.6377**	5.2750	4.9619	**0.7985**	2.2967	2.2275
circularity	**0.0184**	0.0289	0.0352	**0.0007**	0.0025	0.0027	**0.0258**	0.0499	0.0523
narrow_factor	**0.0035**	0.0049	0.0045	**0.0000**	0.0001	0.0001	**0.0052**	0.0112	0.0078
per_dia_ratio	**0.0564**	0.0986	0.1163	**0.0062**	0.0410	0.0402	**0.0787**	0.2024	0.2004
per_length_ratio	**0.0319**	0.0637	0.0768	**0.0021**	0.0212	0.0218	**0.0463**	0.1455	0.1476
per_length_width_ratio	**0.0232**	0.0415	0.0507	**0.0011**	0.0075	0.0078	**0.0330**	0.0865	0.0884

## Data Availability

The dataset used in this study is available at the UBD-herbarium repository (accessed on 27 June 2021).
